# Noise-Optimized CBCT Imaging of Temporomandibular Joints—The Impact of AI on Image Quality

**DOI:** 10.3390/jcm13051502

**Published:** 2024-03-05

**Authors:** Wojciech Kazimierczak, Kamila Kędziora, Joanna Janiszewska-Olszowska, Natalia Kazimierczak, Zbigniew Serafin

**Affiliations:** 1 Department of Radiology and Diagnostic Imaging, Collegium Medicum, Nicolaus Copernicus University in Torun, Jagiellońska 13-15, 85-067 Bydgoszcz, Poland; 2 Department of Interdisciplinary Dentistry, Pomeranian Medical University in Szczecin, 70-111 Szczecin, Poland; 3 Kazimierczak Private Medical Practice, Dworcowa 13/u6a, 85-009 Bydgoszcz, Poland

**Keywords:** cone beam computed tomography, deep learning model, image quality, noise reduction, dental imaging, temporomandibular joint, degenerative joint disease

## Abstract

**Background:** Temporomandibular joint disorder (TMD) is a common medical condition. Cone beam computed tomography (CBCT) is effective in assessing TMD-related bone changes, but image noise may impair diagnosis. Emerging deep learning reconstruction algorithms (DLRs) could minimize noise and improve CBCT image clarity. This study compares standard and deep learning-enhanced CBCT images for image quality in detecting osteoarthritis-related degeneration in TMJs (temporomandibular joints). This study analyzed CBCT images of patients with suspected temporomandibular joint degenerative joint disease (TMJ DJD). **Methods:** The DLM reconstructions were performed with ClariCT.AI software. Image quality was evaluated objectively via CNR in target areas and subjectively by two experts using a five-point scale. Both readers also assessed TMJ DJD lesions. The study involved 50 patients with a mean age of 28.29 years. **Results:** Objective analysis revealed a significantly better image quality in DLM reconstructions (CNR levels; *p* < 0.001). Subjective assessment showed high inter-reader agreement (κ = 0.805) but no significant difference in image quality between the reconstruction types (*p* = 0.055). Lesion counts were not significantly correlated with the reconstruction type (*p* > 0.05). **Conclusions:** The analyzed DLM reconstruction notably enhanced the objective image quality in TMJ CBCT images but did not significantly alter the subjective quality or DJD lesion diagnosis. However, the readers favored DLM images, indicating the potential for better TMD diagnosis with CBCT, meriting more study.

## 1. Introduction

The temporomandibular joint (TMJ) is one of the most complex joints in the human body and is responsible for mastication and speech. Temporomandibular joint disorder (TMD) is a generic term that encompasses various problems related to the TMJ, including issues with the jaw, joints, and masticatory muscles [1]. TMD manifests as tenderness in the muscles and/or TMJ upon palpation, limitations or alterations in the movement of the mandible, TMJ sounds, and pain in the temporomandibular area [2]. The reported prevalence of TMD varies significantly depending on the criteria and the population studied [3,4]. It is estimated that among patients seeking orthodontic treatment, the prevalence of TMD ranges from 21.1% to 73.3% [5]. The etiology of TMD is multifactorial and includes biopsychosocial factors, trauma, malocclusion, and genetic factors [6].

Imaging plays a crucial role in the diagnosis of TMD, and the 2014 guidelines for the diagnostic criteria of TMD stipulate that positive imaging findings from MRI and CT examinations are required for a definitive diagnosis of degenerative joint disease (DJD) and disc displacement (DD), respectively [7]. A significant proportion of TMD cases can be diagnosed and monitored using diagnostic imaging [8]. DJD can be a manifestation of a heterogeneous group of disorders with similar radiological TMJ manifestations: osteoarthritis (OA), rheumatoid arthritis (RA), juvenile idiopathic arthritis (JIA), and spondyloarthropathies [3]. The disease course associated with damage to the cartilage, subchondral bone, and synovial membranes leads to the deterioration of articular cartilage, joint remodeling, and abrasion [3]. Although MRI is considered the gold standard for TMD diagnostics [9], cone beam computed tomography (CBCT) plays an important role in the evaluation of changes in the bone [10,11,12,13]. Due to its lower accessibility and high costs, MRI continues to be the modality reserved for cases with diagnostic difficulties or when the results of imaging may influence the treatment and prognosis of the patient [8]. CBCT has been found to be highly accurate and superior to MRI in assessing the morphology of osseous joint components and cortical bone integrity [10,14]. Therefore, it remains the modality of choice in the assessment of cortical bone integrity due to its multiplanar reformation capabilities and high spatial resolution [10]. 

CBCT has become an important tool in dental imaging, offering precise three-dimensional (3D) images of the dentomaxillofacial area and overcoming the constraints of 2D imaging. With a resolution under 100 µm, CBCT has been widely used for implant planning, periodontics, TMJ imaging, orthodontics, and maxillofacial surgery since its commercial introduction in the 2000s [15]. Compared with CT, CBCT provides precise diagnostics with a lower radiation dose, shorter exposure time, and higher spatial resolution [10,16]. However, CBCT has limitations, such as artifacts and noise in patient images. Noise can hinder the clarity of low-density tissue differentiation, potentially leading to misdiagnoses [17,18]. Because noise is inversely related to the radiation dose, noise-minimizing techniques can lower radiation exposure and enhance CBCT’s diagnostic precision [19].

The most commonly utilized technique for noise optimization in CT is iterative reconstruction (IR). IR has already proven its diagnostic value in both conventional CT [20,21,22,23] and CBCT [24,25,26,27]. Recent advancements in the field of artificial intelligence (AI) have led to the development of deep-learning-based image reconstruction algorithms (DLRs) as an alternative. DLRs have already demonstrated improved diagnostic accuracy with less noise and lower radiation doses [28,29,30]. However, their compatibility is often limited to specific CT scanner vendors, such as GE Healthcare’s TrueFidelity™ or Canon Medical Systems’ AiCE, restricting their use with equipment from other manufacturers. A promising alternative is a vendor-neutral deep learning model (DLM) like ClariCT.AI, which operates in the image post-processing stage without needing crude data. ClariCT.AI, FDA-cleared in 2019, has been shown to reduce noise and maintain diagnostic accuracy on par with vendor-specific DLRs. The algorithm was trained on a dataset of over a million CT images from various vendors and reconstruction settings [31]. Studies by Nam et al. and Park et al. have confirmed ClariCT. AI’s ability to improve the quality and spatial resolution of images, even with a 70% reduction in the radiation dose [31,32]. These findings were later accompanied by other papers showing the high diagnostic value of DLM reconstructions [33,34,35,36,37,38]. Hypothetically, they could also positively affect the quality parameters of TMJ CBCT images, thereby increasing their diagnostic value in the evaluation of DJD-associated lesions. To the best of our knowledge, no studies have analyzed the image quality parameters and diagnostic accuracy of DLM reconstruction in CBCT TMJ evaluation. 

The first aim of this study was to assess the objective and subjective image quality parameters of standard CBCT and DLM-reconstructed TMJ images. The second aim was to compare the detectability of TMJ DJD lesions in standard CBCT images and those reconstructed using DLM. 

## 2. Materials and Methods

### 2.1. Patients

The study population consisted of 53 patients (15 males and 38 females, aged 18–56). All CBCT scans were acquired at a single private orthodontic center. All patients were referred for TMJ CBCT by orthodontists and dental surgeons between January and December 2023. The primary indication for CBCT imaging and inclusion criterion were symptoms of TMD and suspicion of TMJ OA. The main exclusion criterion was the presence of severe motion artifacts. 

### 2.2. Image Acquisition and Post-Processing

All scans were performed using a Hyperion X9 PRO 13 × 10 (MyRay, Imola, Italy). One standard, marked as “Regular”, setting of the apparatus was used with factory TMJ preset (90 KV, 36 mAs, CTDI/Vol 4.09 mGy and 13 cm field of view). According to the apparatus manual, both TMJs were scanned separately. All images were reconstructed at a slice thickness of 0.3 mm. After scanning, the images were anonymized and exported for further analysis. The deep learning, denoised reconstructions were obtained using commercially available DLM (ClariCT.AI—ClariPI, Seoul, Republic of Korea). 

### 2.3. Objective Image Quality

To assess the objective image quality, the radiographer (KK) placed square regions of interest (ROIs) at:

1. Mandibular condyle.
2. TMJ articular space.
3. Masseter muscle.
4. Buccal adipose tissue.

All ROIs were carefully placed in homogenous tissues, avoiding any lesions, inhomogeneities and artifacts. The contrast-to-noise ratio (CNR) was evaluated using ImageJ software v. 1.41 (National Institutes of Health, Bethesda, MD, USA). The ROIs were automatically propagated between the native and DLM reconstructions to maximize the objectivity of the results. The CNR formula was based on the formula presented by Koivisto [39] and calculated as follows:

CNR = (S_R1–2_ − S_M_)/N

where S_R1–2_ is the mean signal at the mandibular condyle and TMJ is the articular space, S_M_ is the mean signal in the background (masseter muscle), and N is the average standard deviation (SD) in the anatomical landmark and background ROI (buccal adipose tissue). 

The CNR values of the ROI_1_ and ROI_2_ in the DLM and native reconstructions were compared to evaluate the effectiveness of the AI denoising tool. 

### 2.4. Subjective Image Quality

The subjective image quality of the overall image quality was assessed by a radiologist and orthodontist (both readers with >5 years of experience in craniofacial CT assessment) who were blinded to patient details and the use of the AI denoising tool. The images were evaluated on a five-point scale (1 = poor, 5 = excellent), considering factors such as noise, sharpness, and the visibility of the anatomical structures of TMJs as follows:

1. anatomical structures not identifiable, images with no diagnostic value;
2. structures identifiable with adequate image quality;
3. anatomical structures still fully assessable in all parts and acceptable image quality;
4. clear delineation of structures and good image quality;
5. excellent delineation of structures and excellent image quality.

Subjective image quality analysis was performed on a dedicated console, the iRYS Viewer. The window width and center were predefined at 1048 and 4096 HU, respectively.

### 2.5. Lesion Assessment

Each of the CT scans was assessed by both of the readers for the presence of the following TMJ DJD characteristics of the mandibular condyle [40]:

- flattening—the loss of the convex form of the articular surface;
- erosion and subchondral cysts—the loss of continuity in the cortical bone margins +/− cavities below the articular surface;
- osteophytes—marginal hypertrophy with sclerotic borders and the exophytic angular formation of the osseous tissue arising from the surface;
- subcortical sclerosis—an increase in the thickness of the cortical plate;
- condylar deformation—abnormal morphology of the condyle.

### 2.6. Inter-Rater Reliability Analysis

To evaluate the reliability of the qualitative assessments performed by the two readers, the agreement between the results of the subjective image quality assessments was calculated.

### 2.7. Error Study

Ten randomly selected subjects (40 CBCT scans) were re-examined by the same author one month after the initial analysis. The ICC for the subjective image quality analyses was calculated to assess the intra-rater agreement.

### 2.8. Statistical Evaluation

The mean, standard deviation, median, quartiles, and range of quantitative variables were calculated. The Mann–Whitney test was used for comparisons of the quantitative variables between two groups. The Chi-squared test (with Yates correction for 2 × 2 tables) or Fisher exact test (in case of low expected values) were used for comparisons of the qualitative variables between groups. The inter-rater reliability of the qualitative measures between two raters was assessed with Cohen kappa. The significance level was set to 0.05. All analyses were conducted using R software version 4.3.2.

## 3. Results

### 3.1. Population, Sample Size

The authors reviewed the CBCT scans of 53 patients. Three patients were excluded because of severe motion artifacts. The CBCT scans of 50 patients were included. In total, 200 CBCT examinations were evaluated by both readers (separately, right and left joint, DLM and native reconstruction). The mean age of all participants was 28.29 years (SD 11.1; median 28; range 11–56). This constituted 37 females with a mean age of 27.23 (SD 11.23; range, 11–56) and 13 males with a mean age of 31.33 (SD 10.68; range, 12–43).

The sample size was confirmed using an online sample size calculator (https://clincalc.com, accessed on 24 January 2024). To investigate the adequacy of our study group’s size, we evaluated the minimal statistically significant differences in the mean CNR values for ROI_1–2_. The obtained mean deviation for ROI_1–2_ was 4.23, which was significantly higher than the calculated minimal significant value of 3.56.

### 3.2. Objective Image Quality

**Figure 1:**
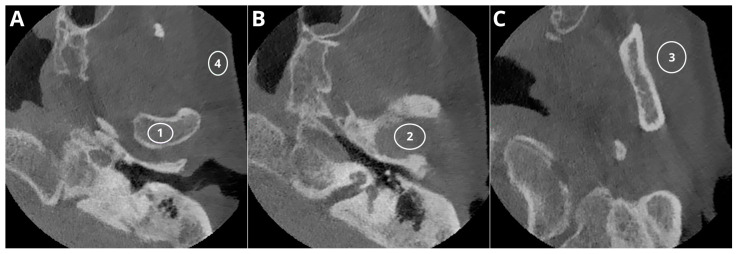
Sample ROI positioning: ROI_1_—condyle, ROI_4_—buccal adipose tissue (**A**); ROI_2_—articular space (**B**); ROI_3_—masseter muscle (**C**).

Table 1 summarizes the results of the objective image quality assessment. Figure 1 shows the sample ROI positioning.

**Figure 2:**
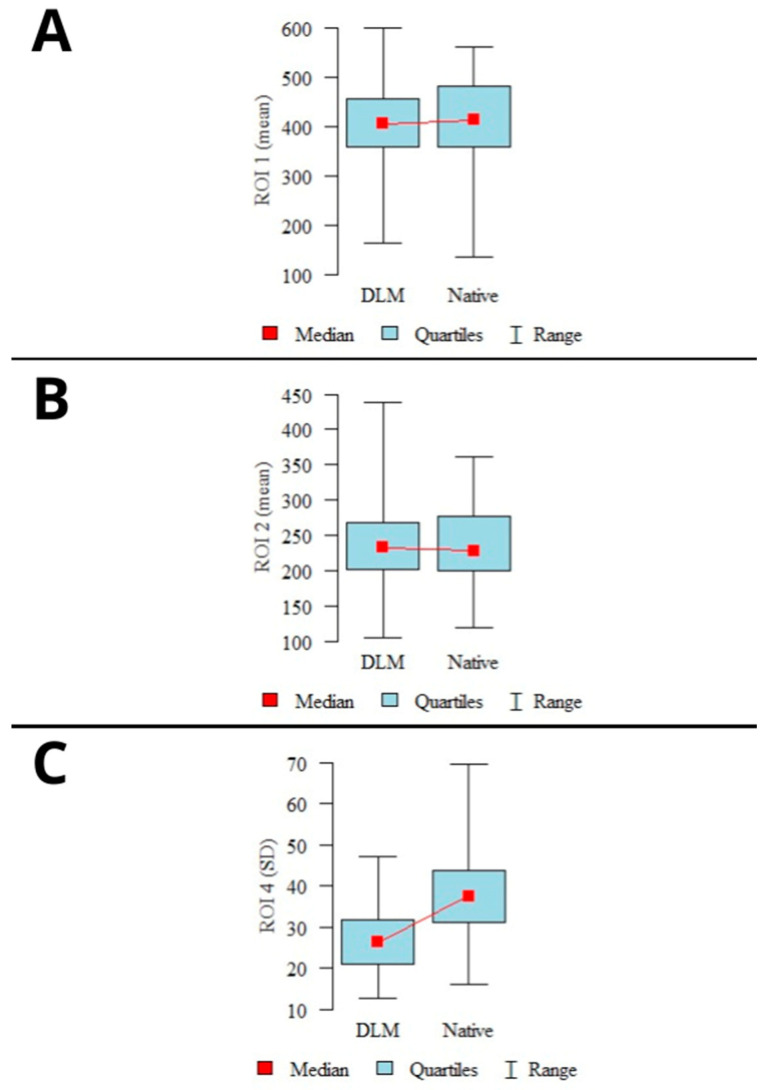
Results of mean signal calculations in ROI_1_ (**A**), ROI_2_ (**B**), and mean noise calculations (**C**). (*mean values, 95% confidence intervals (CI), ranges*).

The average signal measured in ROI_1–2_ showed slightly higher mean values in the native reconstructions than in the DLM images. However, the difference was not statistically significant (*p* > 0.05). The image noise defined as SD in buccal adipose tissue was significantly higher in the native reconstructions (*p* < 0.001). The graphical representation of the mean signal and noise calculations are shown in Figure 2.

**Figure 3:**
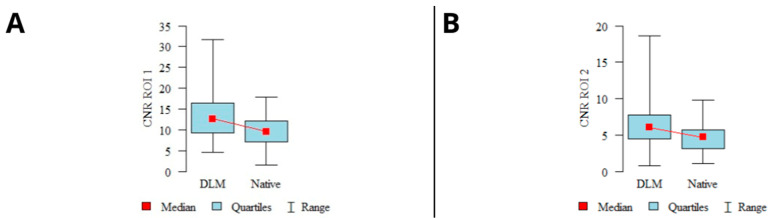
Results of CNR calculations in mandibular condyles (**A**), articular spaces (**B**) (*mean values, 95% confidence intervals (CI), ranges*).

The calculated CNR levels showed a statistically significant difference (*p* < 0.001) for both examined locations (ROI_1–2_), with higher CNR levels observed in the DLM reconstructions. A graphical representation of the CNR calculation results is shown in Figure 3.

### 3.3. Subjective Image Quality

**Figure 4:**
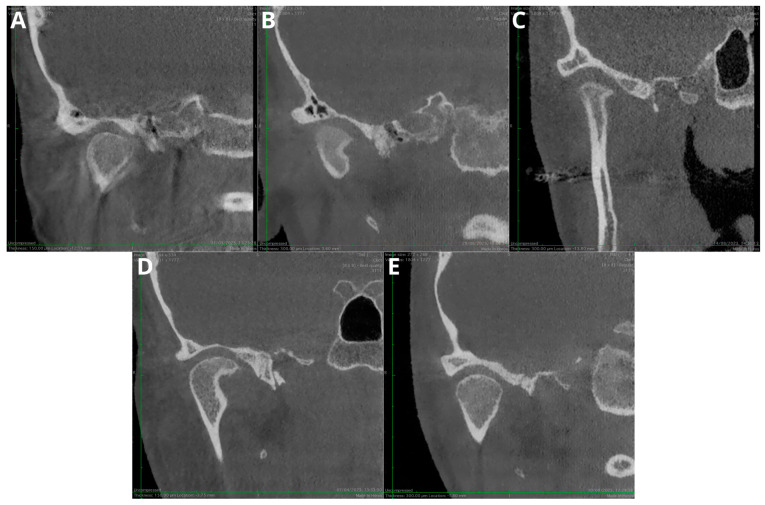
Qualitative image analysis: (**A**)—(1 point) anatomical structures not identifiable and images of no diagnostic value; (**B**)—(2 points) structures identifiable in adequate image quality; (**C**)—(3 points) anatomical structures still fully assessable in all parts and acceptable image quality; (**D**)—(4 points) clear delineation of structures and good image quality; (**E**)—(5 points) excellent delineation of structures and excellent image quality.

Figure 4 presents the exemplary images evaluated according to the 5-point subjective image quality scale; however, the differences were subtle and fully recognizable during CBCT evaluation.

**Figure 5:**
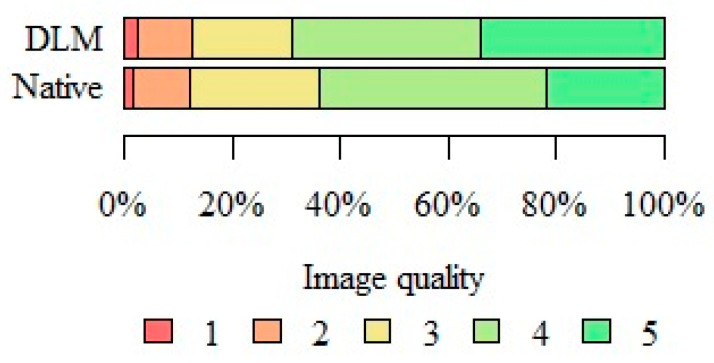
Summary of subjective image quality assessments performed by both readers.

Table 2 presents the summarized results of the subjective image quality assessment. In summary, both readers marked DLM reconstructions with a higher score more often; however, the differences were not statistically significant (*p* = 0.055). The type of reconstruction had no statistically significant impact on the subjective image quality assessment. Figure 5 presents the summarized image quality assessments of both types of reconstruction.

### 3.4. Lesion Assessment

**Figure 6:**
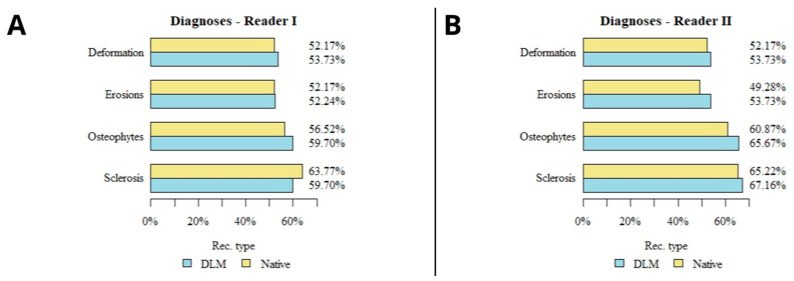
Diagram presenting the results of lesions detected in both reconstructions by both readers (**A**,**B**).

**Figure 7:**
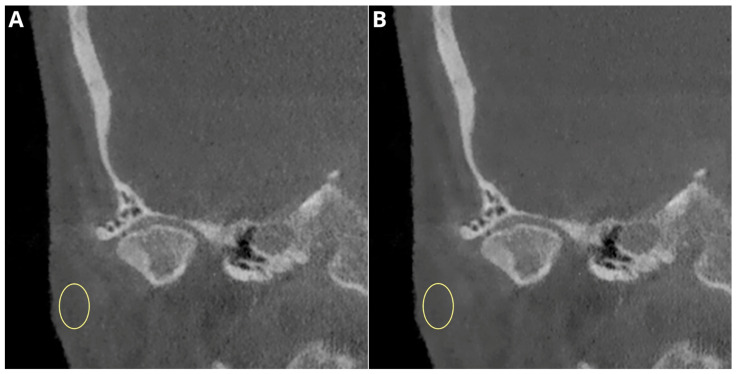
Sample patient diagnosed with erosions, oteophytes, condyle flattening and deformation. Circular ROIs placed in adipose tissue. Reconstructions: (**A**) Native—mean signal −36.6 noise 49.7; (**B**) DLM—mean signal −39.8, noise 43.2.

The results of the subjective image quality assessments for temporomandibular joint TMJ DJD manifestations are summarized in Table 3 and presented in Figure 6. Images with unacceptable quality were excluded from the analysis. No significant correlation was found between the number of lesions diagnosed and the type of reconstruction in the evaluations of both readers. The use of deep-learning-based reconstructions did not significantly affect the assessment of DJD in the TMJ. A schematic graphical representation of the summarized DJD lesions diagnosed by both readers is shown in Figure 6. A sample comparison of the native and DLM reconstructions with the full spectrum of assessed lesions is shown in Figure 7.

### 3.5. Inter-Rater Reliability Analysis

The inter-reader agreement for the subjective image quality of both reconstructions, expressed as Cohen’s kappa, showed strong agreement for all ratings (κ = 0.805). The detailed results of the inter-reader reliability assessment are shown in Table 4.

### 3.6. Error Study

The analysis of the repeatability of the subjective image quality analysis carried out by the reader demonstrated excellent concordance (ICC = 0.833).

## 4. Discussion

Our study aimed to evaluate the impact of DLM reconstruction on the image quality and detectability of lesions in CBCT images of the TMJs. Our findings suggest that while DLM reconstruction significantly improves the objective image quality parameters, the subjective image quality and DJD lesion detectability showed no significant difference compared to standard reconstructions.

The improved objective image quality in DLM reconstructions, as evidenced by higher CNR values, is consistent with previous studies highlighting the potential of AI to enhance image clarity [41]. The reduction in noise without compromising image detail is particularly valuable in TMJ imaging, where small osseous changes can indicate early stages of pathological processes [10]. Despite the objective improvements, subjective assessments did not show a significant preference for DLM reconstructions. This could be due to the subjective nature of image interpretation, where different readers may prioritize different aspects of image quality. Furthermore, the strong inter-reader agreement (κ = 0.805) indicates that the subjective image quality is consistent between readers, and that the preference for DLM may not be universal among radiologists and orthodontists.

We also found that the type of reconstruction did not significantly influence the diagnostic assessment of DJD lesions. This could suggest that current CBCT imaging technology, even without DLM reconstruction, is adequate for the identification of DJD-related changes in TMJs. However, it is important to note that the lack of significant difference in the lesion detectability may also reflect the limitations of CBCT in visualizing early or subtle changes in TMJ tissues that are not primarily osseous. MRI should serve as the first-choice modality when inflammatory changes and soft-tissue pathology are suspected [10,42].

The literature concerning noise optimization in dentomaxillofacial CBCT examinations is very limited. In 2023, a CBCT study by Ramage et al. [27] assessed the effect of standard filtered back projection (FBP) and iterative reconstruction (IR) on image noise. The authors showed that IR significantly reduced the image noise compared to standard FBP images (99.84 ± 16.28 and 198.65 ± 55.58, respectively). Some other studies [43,44,45] have evaluated the effectiveness of generative AI in reducing image noise and metal artifacts in dentomaxillofacial CT images. The studies evaluated the performance of various AI models with Wasserstein loss function (WGAN). Hegazy et al. (2020) [43] improved the image quality of low-dose dental CT scans using a WGAN, despite over-smoothing the small anatomical details. Their 2021 study [45] found that variations in WGANs enhanced the image quality and reduced noise in half-scan CTs. Hu et al. [44] also used a WGAN to effectively reduce noise and artifacts in low-dose dental CTs, surpassing other methods like general GANs and CNNs.

The topic of AI noise optimization in dentomaxillofacial CBCT has still not been sufficiently explored, with no studies published on this topic to date (January 2024). Future research with diverse devices and protocols may reveal clearer distinctions in image quality assessments. Our findings indicate that while quantitative improvement is evident, significant qualitative enhancement may require more stringent criteria. It is likely that the results are close to those published in studies performed using low-radiation-dose protocols in standard CT examinations [31,32,33,34,35,36,37]. DLR has already demonstrated its ability to maintain both subjective and objective image quality parameters even when the radiation dose is reduced by 30–71% compared to standard and iterative reconstructions [41]. However, additional studies using low-dose CBCT protocols are essential to achieve comparable results. The 2020 study by Iskanderani et al. [46] is particularly noteworthy. The researchers conducted standard and low-dose CBCT protocols on the TMJs in a group of 34 patients. The low-dose images were then reconstructed using a standard and noise-optimized protocol. The anatomical visibility and image quality of the TMJ in both low-dose protocols were found to be on par with the default protocol, showing no significant radiographic differences. The average Area Under the Curve (AUC) values were 0.931 for the low-dose and 0.941 for the processed protocols. However, the methodology of the study, which involved two sequential scans of TMJs without medical indications, has raised some serious ethical concerns [47]. Conflicting results were presented in a study by de Oliveira Reis, where the authors evaluated the impact of low-dose CBCT protocols on the visualization of TMJ condylar morphological alterations in dry skulls [48]. According to the study, erosion was over-diagnosed in protocols with larger voxel sizes, and the detection of osteophytes was more accurate in images with smaller voxel sizes. These divergent findings highlight the need for further research in the area of low-dose CBCT TMJ protocols and point towards a promising direction for future studies using noise-optimizing AI tools.

Our study has several strengths, including the use of both objective and subjective measures of image quality and the assessment of the detectability of DJD lesions. Additionally, the use of ClariCT.AI as a vendor-agnostic DLM allows for the potential applicability of our findings across different CBCT systems. However, limitations include the retrospective design and the relatively small sample size. While we achieved high inter-reader agreement, increasing the number of readers or including readers with varying levels of experience could provide additional insights into the clinical applicability of DLM reconstructions. Additionally, we evaluated images acquired only with a “regular quality” preset with a standard radiation dose; therefore, our findings cannot be extrapolated to other protocols. Furthermore, our study has not assessed the standard diagnostic performance parameters, such as sensitivity and specificity.

## 5. Conclusions

In conclusion, our study suggests that DLM reconstruction using ClariCT.AI improves the objective image quality of CBCT images of the TMJ. While the subjective image quality and detectability of DJD lesions were not significantly different from standard reconstructions, the preference of readers for DLM images indicates potential benefits that could enhance the diagnostic capabilities in TMD evaluation. Further research is warranted to explore the potential effect of DLM reconstructions on the diagnostic performance and clinical significance of these findings in a broader context.

## Figures and Tables

**Table 1:** Results of objective image quality assessment.

Parameter	Reconstruction	N	Mean	SD	Median	Min	Max	Q1	Q3	*p*
ROI_1_ (condyle)	DLM	100	401.55	79.27	405.52	163.38	598.78	357.80	454.76	*p* = 0.497
	Native	100	408.45	85.94	412.24	137.07	559.88	357.85	481.42	
ROI_2_ (articular space)	DLM	100	234.98	58.17	232.68	106.34	437.79	201.60	268.17	*p* = 0.752
	Native	100	235.57	51.61	228.49	119.17	361.57	200.13	277.12	
Noise (SD)	DLM	100	27.33	7.39	26.37	12.91	47.26	21.12	31.79	*p* < 0.001 *
	Native	100	37.99	9.09	37.58	16.17	69.58	31.14	43.91	
CNR ROI_1_	DLM	100	13.19	5.16	12.72	4.72	31.66	9.34	16.51	*p* < 0.001 *
	Native	100	9.74	3.52	9.67	1.64	17.93	7.22	12.10	
CNR ROI_2_	DLM	100	6.64	3.35	6.08	0.86	18.67	4.52	7.78	*p* < 0.001 *
	Native	100	4.88	2.04	4.76	1.18	9.78	3.21	5.78	

DLM—deep learning model, N—number, *p*—Mann–Whitney test, SD—standard deviation, Q1—lower quartile, Q3—upper quartile, ROI—region of interest, CNR—contrast to noise ratio. * statistically significant (*p* < 0.05).

**Table 2:** Results of subjective image quality assessment.

Image Quality	Reconstruction Type		*p*
	DLM (N = 200)	Native (N = 200)	
1	4 (2.0%)	2 (1.0%)	*p* = 0.055
2	20 (10.0%)	20 (10.0%)	
3	37 (18.5%)	48 (24.00%)	
4	71 (35.0%)	86 (43.0%)	
5	68 (34.0%)	44 (22.0%)	

DLM—deep learning model, N—number, *p*—Fisher’s exact test.

**Table 3:** Summary of diagnosed DJD lesions.

	Lesion	Reconstruction		*p*
		DLM	Native	
Reader I	Sclerosis	40	44	*p* = 0.755
	Osteophytes	40	39	*p* = 0.84
	Erosions	35	36	*p* = 1
	Deformation	36	36	*p* = 0.992
Reader II	Sclerosis	45	45	*p* = 0.953
	Osteophytes	44	42	*p* = 0.687
	Erosions	36	34	*p* = 0.728
	Deformation	36	36	*p* = 0.992

DLM—deep learning model, *p*—Mann–Whitney test.

**Table 4:** Results of inter-reader agreement assessment.

κ	95% CI		Agreement	Interpretation
0.805	0.738	0.871	85.99%	Strong

## Data Availability

Data available upon request.
